# Early Basal Cuspal Tear of a Porcine Bioprosthetic Mitral Valve Causing Massive Mitral Regurgitation

**DOI:** 10.3390/jcdd7040052

**Published:** 2020-11-06

**Authors:** Muhammed Tamim, Christos Alexiou, Yaser AlKadi, Mohsen S. Mahmoud, Fatema Qaddoura

**Affiliations:** 1 Cardiac Surgery Department, King Fahd Military Medical Complex, Dhahran 31932, Saudi Arabia; alexiou486@aol.com (C.A.); yasserkady@hotmail.com (Y.A.); 2 Department of Critical Care Medicine, Cairo University Kasr Alaini, Cairo 11562, Egypt; mohsensalah70@hotmail.com; 3 Department of cardiology, King Fahd Military Medical Complex, Dhahran 31932, Saudi Arabia; qaddoura61@yahoo.com (F.Q.)

**Keywords:** mitral valve, bioprosthesis dysfunction, leaflet tear

## Abstract

Although porcine mitral bioprostheses provide predictably good long-term outcomes, unexpected leaflet tears leading to abrupt haemodynamic changes may occur. Here, we report on a patient who was presented with acute dyspnea due to a cuspal tear of a porcine bioprosthetic mitral valve causing severe mitral regurgitation. Her condition was subsequently complicated by a systemic infection, probably pneumonia, and was successfully managed with an urgent redo-mitral valve replacement.

## 1. Introduction

An appropriate follow-up after surgical implantation of bioprostheses ensures the timely detection of dysfunctional heart valves and their replacement in a safe manner [1,2]. Nonetheless, catastrophic structural valve deterioration can occur [1,2,3,4,5] early or late postoperatively [6,7]. We describe the clinical presentation, management, and outcome of a patient who suffered an early cuspal tear of a bioprosthetic mitral valve 4 years post valve implantation and discuss her case in context with the pre-existing relevant literature.

## 2. Case History

The paper was conducted in accordance with the Declaration of Helsinki, and the case report was approved by the Ethics Committee of Education and Training Department at King Fahd Military Medical Complex, Dhahran (Approval No: KFMMC-REC-2016-12/14). A 53-year old female patient was admitted in a peripheral hospital with severe, acute onset dyspnea and orthopnea. Her past medical history included a bioprosthetic aortic valve replacement (AVR) and mitral valve replacement (MVR) 4 years ago (Mosaic, Medtronic Minneapolis, MN, USA). Two hours later, she required intubation and mechanical ventilation, and transthoracic ECHO showed significant MR. Two days later, she developed a high-grade fever (39 °C), accompanied by radiological changes compatible with a right lower lobe pneumonia, and a sharp rise in serum inflammatory markers. Multiple blood cultures, skin and nasopharyngeal swabs were taken, and she was commenced on i.v. antibiotics (Tamiflu, Tazocin, Azithromycin). All cultures returned negative including those for MERS-COV and H1N1. Meanwhile, she grew *Methicillin Resistant Staphylococcus Aureus* in a nasopharyngeal swab and the antibiotic treatment was changed to i.v. Meropenem, Vancomycin, and Levofloxacin. The transesophageal ECHO (TEE) showed severe MR and malfunction of the bioprosthetic MV and was referred to our center.

**Figure 1:**
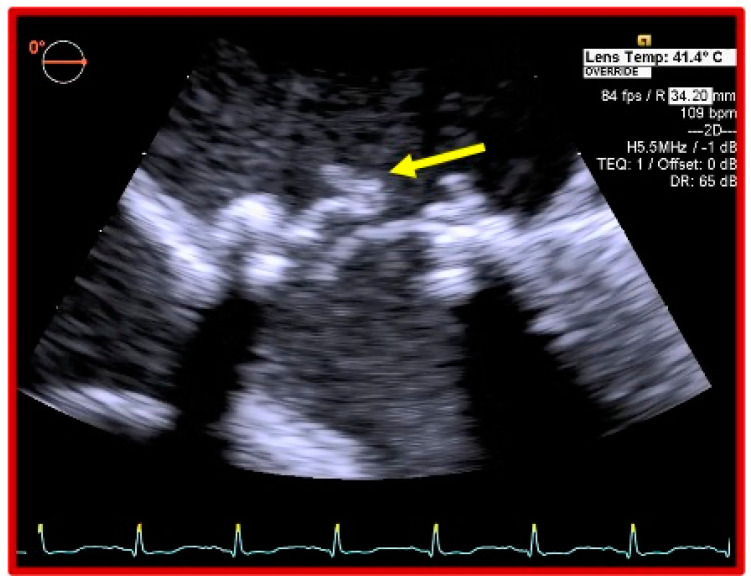
Two-dimensional transesophageal ECHO showing a flail leaflet of the bioprosthetic mitral valve.

**Figure 2:**
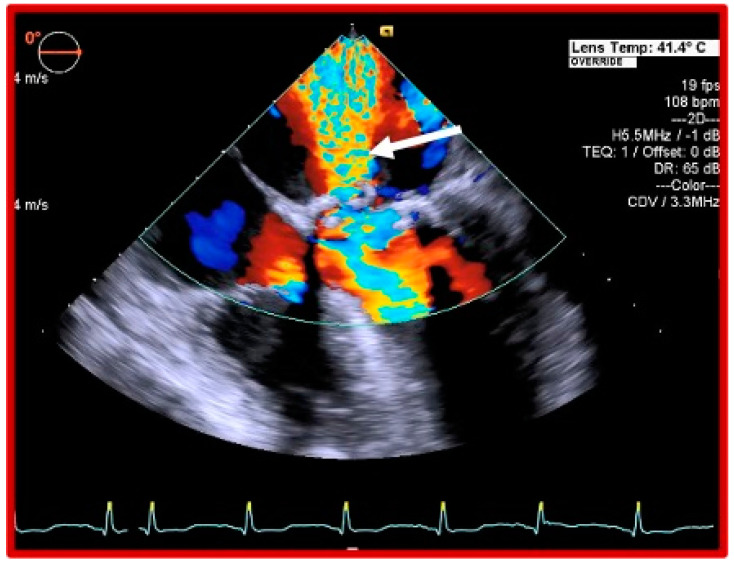
Doppler two-dimensional (2D) transesophageal ECHO showing a large mitral regurgitant jet.

**Figure 3:**
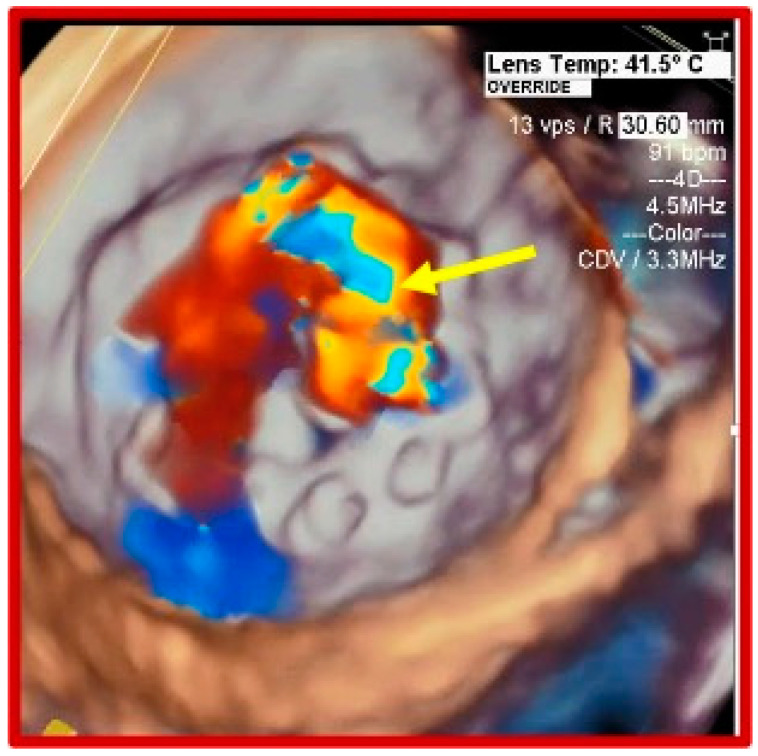
Three-dimensional color doppler transesophageal ECHO showing a large mitral regurgitant get.

On admission to the intensive care unit, she was mechanically ventilated, highly pyrexial, on high doses of noradrenaline. A repeat TEE showed a flail mitral valve leaflet and severe MR (Figure 1), 2D TEE (Figure 2), and 3D TEE (Figure 3), dilated left atrium (LA), elevated systolic pulmonary artery pressure (75 mmHg), moderate tricuspid regurgitation (TR), accelerated flow across the aortic bioprosthesis (mean gradient 32 mmHg), and preserved bi-ventricular function. A computed tomography of the chest and abdomen showed a right lower lobe consolidation consistent with pneumonia. She continued to run a high fever and to require high doses of vasopressors and inotropes in order to maintain an adequate blood pressure and urine output. Considering the persisting fever, the unsatisfactory response to antibiotic therapy, severe haemodynamic compromise caused by the mechanical dysfunction of the prosthetic MV, and the risk for irreversible multiorgan failure, a decision was taken to proceed with an urgent surgical intervention 7 days after her admission to our hospital.

**Figure 4:**
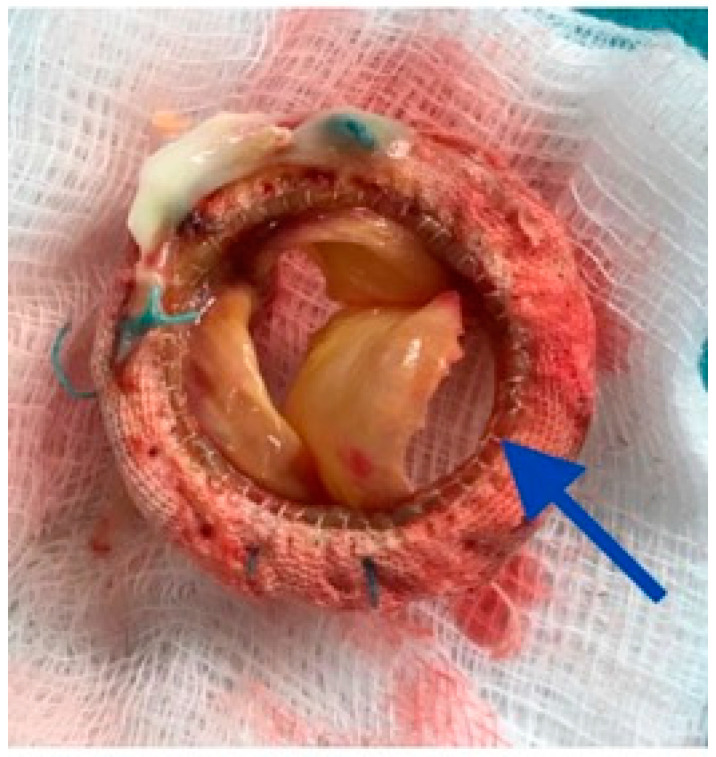
Explanted mitral valve bioprosthesis basal cusp tear (blue arrow) almost from commissure to commissure.

A standard redo-MVR and De Vega TV annuloplasty were performed through a median re-sternotomy, utilizing cardiopulmonary bypass (aortic and bicaval cannulation), antegrade delivery of Del Nido cardioplegia, and a right atrial-transeptal route to access LA and the MV. On inspection, one of the leaflets of the prosthetic MV was detached across its base from the frame of the valve, almost from commissure to commissure (Figure 4). The valve otherwise appeared normal without vegetations, thrombus, or signs of valve dehiscence. The bioprosthesis was excised and after thorough tissue debridement and washing with normal saline, a mechanical valve (27 mm On-XCryoLife, Inc., Kennesaw, NW, USA) was implanted. The LA appendage was obliterated with continuous suture, the interatrial septum closed. The aortic valve was inspected through a small aortotomy, which revealed a well-functioning valve without any vegetation and the operation was completed in the standard manner. An intraoperative TEE showed moderate biventricular dysfunction, well seating and normally functioning prosthetic MV without paravalvular leak, and a mild TR.

Six days after surgery, the inotropes were stopped, the patient became afebrile, and was extubated. Thereafter, she made steady progress becoming fully ambulant; Warfarin was commenced aiming for an INR of 2.5–3. A culture of the explanted valve was negative as were the tissues and fluids taken at operation. The patient was eventually transferred to the referring hospital 3 weeks after her operation for convalescence and completion of her antibiotic therapy. The pre-discharge ECHO showed well-functioning valves, mild TR, and a mildly impaired bi-ventricular function.

## 3. Discussion

Bioprosthetic valves have good durability and a predictable mode of failure, with gradual degeneration of the leaflet tissue being the norm [1,2,3]. As a result, when a re-operation is required, it is usually done in a controlled and safe manner [1,2,3]. Nevertheless, some patients do experience sudden valve dysfunction due to acute structural valve deterioration, needing urgent attention [1,2,3,6,7].

In a cohort of 836 patients who had a porcine bioprosthetic valve replacement, 28 required a re-operation with 18 of them having a leaflet tear of the explanted valve. Of these 18 patients, 12 had a previous MVR and six had AVR [3]. Thus, the proportion of bioprosthetic valve leaflet tear leading to a re-operation was 2.1% [3]. The cusp rupture or detachment in a mitral bioprosthesis may cause a massive MR with serious consequences [3,4,5,6,7]. Amongst the 12 patients who had a leaflet tear in a mitral bioprosthesis in Pomar’s series, six presented in NYHA III and six in NYHA IV [3].

Although our patient was relatively young at the time of the first operation, she insisted on having bioprosthesis valves, aiming at an anticoagulation-free life as she was an active traveler. The risk of early valve deterioration was explained to her, but we did not anticipate it to occur so fast. The initial diagnosis in our patient was cardiogenic pulmonary oedema, which suggests that the cuspal tear of the bioprosthesis and the ensuing massive MR was the primary event. Infective endocarditis was excluded subsequently by laboratory and echocardiographical findings 

In either case, considering the major hemodynamic burden caused by the massive MR, an urgent surgical intervention was imperative for our patient [8]. Notably, all six patients who had a cuspal tear of a bioprosthetic MV went on to have a redo MVR in the elegant study by Pomar [3].

Single or multiple cuspal tears, leaflet perforations, free-edge tears, and basal tears have all been described with the latter (also seen in our patient) being the commonest [3,4,5,6,7]. Some torn leaflets are calcified, whereas in others calcification is visible only under light microscopy [3]. In our patient, the leaflet tear occurred at the base across its attachment onto the frame of the prosthetic valve (Figure 4) bearing no calcium, vegetations, or other signs suggestive of infection, and the valve culture was negative. This implies that the detachment of the leaflet from the frame of the valve was a purely mechanical event [9].

The leaflet detachment occurred 4 years after surgery in our patient, an average time for cuspal tears of around 4–6 years, as quoted by Pomar [3]. However, following an implantation of a mitral bioprosthesis, Miura [6] reported a cusp detachment occurring only 10 months after surgery and Ha et al. [7] 12 years after surgery.

## 4. Conclusions

Although porcine MV bioprostheses provide predictably good long-term outcomes, unexpected cusp tears leading to abrupt haemodynamic changes with disastrous consequences may occur. This reminds us of the need for meticulous clinical and echocardiographical follow-up of these patients.
